# Mitigating COVID-19 Mortality and Morbidity in China's Aging Population: A Focus on Available Medications and Future Developments

**DOI:** 10.14336/AD.2023.0318

**Published:** 2023-12-01

**Authors:** Evelyne Bischof

**Affiliations:** ^1^State Key Laboratory of Oncogenes and Related Genes, Shanghai Cancer Institute, Department of Oncology, Renji Hospital, School of Medicine, Shanghai Jiao Tong University, Shanghai, China.; ^2^Department of Advanced Biomedical Sciences, Federico II University of Naples, Naples, Italy.; ^3^Shanghai University of Medicine and Health Sciences, Shanghai, China.

**Keywords:** mitigating, COVID-19, mortality, morbidity, aging population

## Abstract

The COVID-19 pandemic, often referred to as the geropandemic, has put immense pressure on global healthcare systems worldwide, leading to a rush in the development and approval of medications for the treatment of the viral infection. Clinical trials on efficacy and safety had a limited spectrum on inclusion and endpoints because of the urgent need for fast results. The chronologically and biologically aged population is especially at risk for severe or lethal disease, as well as treatment-associated toxicity. In China, the growing elderly population segment has been a focus in public health measurements of COVID-19, guiding towards herd immunity with a mild variant, thus minimizing overall deaths and morbidity. While the COVID-19 pandemic has now been reclassified and the virus weakened, there is a clear need for novel therapies to protect the elderly. This paper reviews the current safety and efficacy of available COVID-19 medications in China, with a specific focus on 3CL protease inhibitors and the aging population. The current COVID wave in China has demonstrated a significant impact on the elderly and the need for new drugs that are effective at low doses and can be used alone, without harmful side effects, generation of viral resistance, and drug-drug interactions. The rush to develop and approve COVID-19 medications has brought up important questions about the balance between speed and caution, resulting in a pipeline of novel therapies now moving through clinical trials, including third-generation 3CL protease inhibitors. A majority of those therapeutics are being developed in China.

The development of pharmacotherapies against the new SARS-CoV-2 virus since its onset in 2020 prioritized accelerated testing leading to rapid approval for use [1]. Inevitably, there was only marginal focus on extended dosing, duration, safety, and efficacy endpoint measurements and the inclusion of specific populations, such as those at high risk, pregnant, multimorbid, and aged persons [2-4]. Especially the latter group proved to be the most vulnerable and recorded the highest mortality rate. Despite this obvious fact, the currently rapidly approved therapeutics for COVID-19 are not optimized or tested in the elderly. A new generation of core therapeutics, especially 3CL-protease inhibitors, is urgently needed. Exclusion of the elderly in the clinical trials under pressure to recognize therapeutics is accompanied by further deficiencies, e.g., despite quite early insights on sex and gender disparities in COVID-19, many clinical trials did not include those as biovariables [5]. Meanwhile, it has been reported that males are affected more severely by COVID while females have a distinct immune response and are more prone to experience a cytokine storm. Despite that fact, a more targeted optimization of dosing and onset of therapy is of great importance, as the male/female ratio becomes less with increasing age of the groups [6-11].

The initial accelerated approvals of COVID-19 drugs have been critical in ameliorating the evolving pandemic situation. This was particularly important in the early stages where there was only a rudimentary understanding of the virus' specific structure and pathomechanisms [12]. Nevertheless, there is still insufficient data on the safety and efficacy of some of the drugs granted emergency approval, as well as reported sex differences as biovariables [10].

This article aims to address several key research questions and objectives related to COVID-19 and its impact on aging populations, particularly in China. It describes the relationship between COVID-19 and aging populations, highlighting the unique vulnerabilities and risks faced by elderly individuals exposed to SARS-CoV. It opens a discussion of the special need for safe and effective medications for vulnerable elderly populations, emphasizing the importance of developing treatments that are specifically tailored to their unique needs and challenges. It provides an overview of currently available drugs for COVID-19, focusing specifically on their application in China during the most recent surge of infection. Finally, it highlights the development pipeline of 3CL protease inhibitors, which have shown promise as potential treatments for COVID-19.

## Aging and COVID - a vicious circle and China perspective

The COVID-19 pandemic has initiated numerous debates and stimulated research in aging and related domains, while geriatric adjustments have become more pronounced in a variety of sub-disciplines on the frontline, and later in disciplines covering the sequelae of COVID-19 and Long-COVID [13]. The “gero-pandemic” has perpetuated investigations of potential targets of both aging and pathological processes, as well as their convergence [14, 15].

Chronologically older adults, as well as those biologically aged individuals (e.g., due to comorbidities such as oncological or autoimmune diseases, altered microbiome, etc.), are primarily at risk of a lethal or, at best, a chronic protracted course of the disease, especially when there is no supportive a priori mounting of vaccination by geroprotectors [16-18]. Conversely, the infection itself accelerates biological aging via various pathomechanisms, such as inflammation (accelerated inflamm-aging) [19-21]. At the same time, it has been well-documented for over 20 years that influenza-like infections, particularly influenza itself, result in increased cardiopulmonary morbidity and mortality. This is now also very plausible with the ongoing virus circulation of SARS-CoV-2. Thus, a protracted, chronic COVID-19 disease might contribute to accelerated aging. This is a vicious circle that can be broken with safe and effective treatment options.

China is experiencing a typical sociodemographic transition as has been the case in other countries alongside their development processes. This year was special due to a decline in its population growth for the first time in 60 years. At the end of 2022, the population of Mainland China was estimated to be 1.41175 billion, which is 850,000 fewer than at the end of 2021. The annual birth number was 9.56 million, with a birth rate of 6.77 per thousand, while the death rate reached 10.41 million, at a mortality rate of 7.37 per thousand, resulting in a population growth rate of -0.60 per thousand, with a balanced male to female sex ratio of 104.69 [22].

Since 2022, China's elderly population has grown rapidly. According to data released by the National Bureau of Statistics, the newborn population in China was 367 million from 1962 to 1975. It is obvious that these people will grow old in the coming decades.

Nevertheless, the sheer size of China gives it substantial global significance. Governmental incentives might reverse the decline in the birth rate, and the ongoing efforts towards a healthy longevity of citizens contribute to a significantly lower rate of frailty and multimorbid dysfunctionality among those aged above 65 years, a majority of whom do not cease to be professionally engaged long after the legal retirement age. 209.78 million mainland Chinese citizens are 65 years old and over, accounting for 14.9% of the national population, which has increased by 9.22 million, or 0.7%, since 2021.

"While the COVID-19 crisis led to a reshaping of society in various countries, the Chinese public health authorities anticipated and mitigated a potentially significant impact on the vulnerable older adult population to avoid massive negative impacts on physical, psychological, social, and economic levels [15]. Although ageism was not expected, the social isolation and unfavorable chronic health effects experienced by much of its population required a salutogenic strategy, which was executed initially by strict prevention, positive pre-adaptation of people and the healthcare system, and ultimately a herd immunization with less aggressive SARS-CoV-2 variants. In some other parts of the world, hospitalized patients with a SARS-CoV-2 infection received intensive care due to COVID-19 at the beginning of the pandemic, especially for typical pneumonia. In contrast, the situation in China was similar to what occurred in 2021 in the West: a massively reduced mortality and morbidity rate due to vaccinations and hybrid immunity, especially during the emergence of Omicron and its variants.

China has experienced a continuous rise towards becoming one of the global leaders in biotech and innovation healthcare, including drug discovery and related clinical trials of new compounds. In addition, the number of internet hospitals alone is estimated to be almost 2000, delivering smoother access to healthcare for everyone, especially the elderly who are not in metropolitan areas and cannot physically reach a top-level hospital or pharmacy as well as those who are exposed to infection risks at physical healthcare institutions. Again, the elderly are the major part of this group, especially those with critical and complicated conditions [23, 24].

These factors enable China to bring promising COVID-19 therapeutics to the market and make them easily accessible to the masses.

## Healthy Longevity Medicine and Science in China

One of the top priorities of the progressive healthcare policies and public health in the country, which is benefiting massively from a fruitful and evolving biomedtech environment, is healthy longevity. Healthy longevity medicine, defined as the optimization of healthspan along lifespan by targeting aging processes, aims to mitigate and eliminate the risk of developing age-related diseases, which lead to frailty, loss of productivity, and a massive burden on medical resources. Various initiatives have been put in place to accelerate geroscientific research, as well as translation into the clinic and real-world programs. Coordinated efforts are facilitated through Healthy Aging Institutes and the support of Smart and Internet Hospitals.

## The Sequelae of COVID-19 and the need for rapid recovery

One of the core reasons for accelerating pharmacologic solutions to COVID-19, besides the acute disease and reduction of severe conduct of illness, is the increasing knowledge of long-term post-recovery sequelae. Long-COVID and a variety of further solitary post-COVID residual symptoms have been described and recently labeled as a parallel pandemic [25]. Some of the post-COVID cases are multiorgan damage-related debilitating conditions, ranging as far as severe tremors, chronic pain, metabolic disturbances, and serious cognitive impairment. The pathomechanisms are still not fully understood, and effective treatments remain underdeveloped [26].

One of the long-term conditions patients experience after recovering from COVID-19 is a new form of progressive pulmonary fibrosis that causes a severe decrease in lung capacity [27]. This post-COVID pulmonary fibrosis (PCPF) was found in patients who were hospitalized due to COVID-19, but who were not always so seriously ill that they had to be ventilated or suffered lung failure - two well-known risks for the development of pulmonary fibrosis [28, 29]. The atypical disease course for pulmonary fibrosis caused tissue changes only four to twelve weeks after infection. Avoiding hospitalization and providing accelerated recovery is thus of utmost importance to curb the incidence of such cases.

Two hypotheses that address Long-COVID suggest that viral RNA persists in organ tissues and/or that tissue damage occurs during hospitalization following a severe course of infection. A significant reduction in patients’ brain mass was recorded in the UK database, even after a mild infection with COVID.

We need to understand the connection with SARS-CoV-2 more precisely and investigate whether treatment in the acute phase of the disease has an impact on the development of micro- and macroscopic damage in various systems. A large number of patients suffering from "long haul" syndrome are incapable of returning to and performing professional engagements, causing adverse socio-economic impacts. In both hypotheses, antiviral therapies are of major importance to swiftly eliminate the virus from the organisms and effectively and permanently block the replication to avoid Long-COVID, which has affected an estimated 45 million cases worldwide [30-32].

In China, Long-COVID has not yet manifested itself as a nationwide problem, but it is expected to arise. Almost 60% of the COVID-19 patients in Wuhan still have sequalae with increased pain and fatigue symptoms [33-35]. Targeting the disease at its root with effective medication is likely to contribute to preventing Long-COVID.

## Lessons on novel therapies from the COVID-19 wave in China 2023

During the recent wave of COVID infections in China in December 2022 and early 2023, Nirmatrelvir/ritonavir (Paxlovid) was used in selected hospitalized patients with indications, according to the treatment guidelines, in which the drug had already been integrated in early 2022 ("Diagnosis and Treatment Plan for Novel Coronavirus Pneumonia (9th Edition)"). Having a targeted antiviral available during a surge of infections is certainly advantageous. However, it became quite obvious that the clinical use of the drug was severely limited because the majority of hospitalized patients were elderly and multimorbid. Those with renal and hepatic insufficiencies were often treated with a lower dose at the discretion of the treating physicians (with even further reduced efficacy as per clinical observation). Official data on the efficacy of Paxlovid in China is still pending. However, single-center experiences suggest that there is rather diminished expectation that the drug will result in significant improvement in the course of recovery. Nirmatrelvir is an inhibitor of 3CLpro, which can directly bind to the active site, inhibit the activity of the protease, and thus decelerate or cease the replication of the virus. However, the well-known rebound effect has been frequently observed, especially in the vulnerable population of the elderly [35-38]. It is possible that these observations are biased due to the fact that most patients on the therapy were not meeting the actual recommendation of the Drug Administration, i.e., to be initiated as soon as possible within 5 days after the diagnosis and the onset of symptoms, where Paxlovid is suggested for the treatment of mild to moderate COVID-19 pneumonia in adults with high risk factors (this includes age above 60 years) for severe disease. In the real world, however, mostly only severely sick patients receive the drug only several days after the onset of the disease. Further practical considerations in frail elderly patients became apparent, e.g., polypharmacy (attention to CYP3A inducers, CYP3A-dependent meds including antihypertensives and statins), dysphagia (3 pills at the same time), little safety data regarding invulnerable patients prone to adverse reactions, etc[38]. The drug will not be covered by insurance in China beyond March 2023.

**Table 1:** Overview over 3CL protease inhibitors generation 1-3, and their respective country of discovery, highest status of clinical trials and administration routes.

Generation	Molecule	Countries	Active companies	Highest Status	Administration route
The 1st generation 3CLpro inhibitors have to co-administrate with ritonavir; may induce the potential DDI issue and resistance to HIV protease inhibitors	Nirmatrelvir + Ritonavir (Paxlovid)	USA	Pfizer Inc	Emergency Use Authorization in US, EU, Japan and China	Oral
	SIM-0417	CHINA	Jiangsu Simcere Pharmaceutical Co., Ltd	Conditional approval in China	Oral
	GST-HG171	CHINA	Cosunter Pharmaceutical	Phase II/III	Oral
	QLS1128	CHINA	Qilu Pharmaceutical Co Ltd	Phase I	Oral
	ASC-11	CHINA	Ascletis Pharma Inc	Phase I	Oral
The 2nd generation 3CLpro inhibitors reversible inhibition; achieve single agent without ritonavir boost but have poor permeability	Ensitrelvir	JAPAN	Shionogi & Co Ltd	Emergency Use Authorization n Japan	Oral
	RAY1216	CHINA	Guangdong Zhongsheng Ruichuang Pharmaceutical Co Ltd	Phase III	Oral
	FB-2001	CHINA	Frontier Biotechnologies	Phase II/III	Inhalation
	PBI-0451	USA	Pardes Biosciences Inc	Phase II	Oral
	EDP-235	USA	Enanta Pharmaceuticals Inc	Phase II	Oral
	GS221	CHINA	Grand Pharmaceutical Group Ltd	Phase II	Oral
	SYH2055	CHINA	CSPC Pharmaceutical Group Ltd	Phase I	Oral
	SAL0133	CHINA	Salubris Pharmaceuticals	Phase I	Oral
	STI-1558	USA	ACEA pharma	Phase I	Oral
	PF-07817883	USA	Pfizer Inc	Phase I	Oral
The 3rd generation 3CLpro inhibitor Novel single-agent oral broad-spectrum irreversible inhibitors	ISM3312	CHINA	Insilico Medicine	Phase 1	Oral

Domestic scientists conducted a randomized controlled phase III trial of the compound VV116 and Paxlovid, demonstrating non-inferiority regarding time to sustained clinical recovery among adults with mild-to-moderate COVID-19 at risk of progression [39]. The study reported fewer safety concerns in VV116 as it does not interact with drug transporters or metabolism. However, the study’s primary metric was similar to those previously approved by the FDA for antivirals such as Xofluza and Tamiflu, which have been shown to alleviate symptoms faster than alternatives. In contrast, the EPIC-HR (Evaluation of Protease Inhibition for COVID-19 in High-Risk Patients) trial’s primary endpoint for Paxlovid was its effectiveness in preventing hospitalization or death [40]. The results showed an 80% reduction in the risk of hospitalization or death compared to placebo, while also reducing the viral load on day 5 by 10 times. However, randomized trials have not yet studied the speed at which symptoms are alleviated [39]. On December 30, 2021, VV116 was approved for marketing in Uzbekistan. Currently, two placebo-controlled phase III clinical studies are ongoing. Paxlovid did not show any significant benefit in standard risk patients, and the corresponding trial EPIC-SR was terminated.

The State Food and Drug Administration conducted an emergency review and conditionally approved the Class 1 innovative drug Cenotevir/ritonavir (tablets) and Mindevir (Deuterium Remidevir Hydrobromide tablets). The above drugs are oral small-molecule drugs for the treatment of adult patients with mild to moderate novel coronavirus infection (COVID-19). Cenotevir, as the first domestic 3CL protease inhibitor (inhibitor of the RNA-dependent RNA polymerase), has been studied locally and compared with a placebo group. The treatment group had a significantly shortened time to recovery (1.5 days), and the subgroup of the high-risk group of severe illness was significantly shortened by about 2.4 days from the time from the first administration of 11 related symptoms to the complete asymptomatic state. After receiving a complete 5-day course of treatment of 0.750g of Cenotevir (0.375gx2 tablets) combined with 0.1g of ritonavir (0.1gx1 tablet), administered orally every 12 hours, the viral load was significantly reduced by 96%, and the time until testing negative under PCR tests was significantly shortened by 2.2 days. Detailed data from the study and a publication after peer review are awaited, while provisional reimbursement by insurance is available until the end of March 2023.

Mindevir is an oral nucleoside drug that non-covalently binds to the active center of the RNA polymerase and mainly functions as a mutagen by increasing the frequency of transition mutations (G-to-A and C-to-U) in the viral genes. Preclinical studies have shown significant antiviral effects on the original and mutant strains of SARS-CoV-2, including Omicron.

In China, over 30 companies are currently developing and studying oral small molecule drugs for the treatment of SARS-CoV-2. Those targeting 3CL protease inhibitors are listed in Table 1. The country is expanding the types and quantities of new drugs, and all domestic COVID-19 medicines have been added to the national medical reimbursement list. Two newly approved homegrown pills — Xiannuoxin made by Simcere Pharmaceutical Group and VV116 developed by Shanghai Vinnerna Biosciences — are temporarily covered until March 31. Azvudine, the first domestic COVID-19 oral medication developed by Henan Genuine Biotech, was officially included in the latest version of the national reimbursement list released on January 18. Additionally, the administration has added three traditional Chinese medicine drugs and three herbal formulas to the list.

## 3CL-protease inhibitors - three generations

Among the variety of proteases in the SARS-CoV2 virus, 3CL (3CLpro) protease is the main RNA processing protease in the virus’ own genetic material and is a crucial enzyme in the replication cycle of SARS-CoV-2. The mechanisms of action of these inhibitors vary, but they all work by targeting the 3CL protease and inhibiting its activity, which ultimately prevents the virus from replicating. One type of 3CL protease inhibitor is a peptidomimetic inhibitor, which mimics the structure of the natural substrate of the protease. This type of inhibitor binds to the active site of the 3CL protease and blocks its activity, preventing the virus from replicating. Another type of 3CL protease inhibitor is a covalent inhibitor, which forms a chemical bond with the protease and irreversibly inactivates it. This type of inhibitor is highly specific for the 3CL protease and has been shown to be effective in vitro and in animal studies.

A third type of 3CL protease inhibitor is a non-covalent inhibitor, which binds to the protease but does not form a covalent bond. This type of inhibitor's reversibility depends on its structure and mechanism of action.

Overall, the mechanisms of action of 3CL protease inhibitors involve inhibiting the activity of the 3CL protease, which is essential for the replication of coronaviruses, including SARS-CoV-2. By preventing the virus from replicating, these inhibitors have the potential to treat or prevent COVID-19.

Early repurposing and compound discoveries focused on 3CLpro since HIV-specific proteases did not show promising inhibitory results in the early stage of the pandemic [41-44]. The inhibition of 3CL protease results in the disruption of viral replication, and as such, it was considered a core therapeutic target from the early stages of the pandemic, leading to early suggestions for application in the prophylactic settings, as well as a rapid race by various companies toward repurposing existing drugs or the development of novel 3CLpro inhibitors for treatment purposes [45-49].

Several studies have shown that 3CLpro inhibitors can effectively reduce viral loads and improve outcomes in preclinical models of SARS-CoV-1 infection [50]. SARS-CoV-2 has 79.5% sequence identity with SARS-CoV-1 [51]. However, the high mutability of the virus means that resistance to 3CLpro inhibitors emerges quickly, highlighting the need for either early combination therapies and ongoing monitoring of antiviral resistance, or a fully novel generation of protease inhibitors that can overcome the challenges as single agents [52, 53].

The Table 1 summarizes the three generations of 3CLpro inhibitors, their current stage of clinical development, and their route of application. Ten out of 16 protease inhibitors originate from China, five from the USA, and one from Japan. All except FB-2001 (II generation, inhalation) are oral compounds. Oral routes are preferred in general settings, while inhalations and intravenous administration (still not feasible) are reserved for acute care settings, such as intubated patients without nasogastric tubes.

The first-generation compounds are limited by several factors, such as the need for co-administration with ritonavir (or another CYP3A4 inactivator) to maintain the drug plasma concentration [54, 55]. However, such a co-compound may stimulate drug-drug interactions in clinical usage. Ritonavir is contraindicated to be used concomitantly with a wide range of drugs that are highly dependent on CYP3A4 for clearance, and for which elevated concentrations are associated with severe adverse reactions, such as pulmonary arterial hypertension, oncological, and arrhythmic medications [56-61]. At the same time, co-administration with potent CYP3A4 inducers is also problematic, since significantly reduced 3CLpro inhibitors or ritonavir plasma concentrations may be associated with the potential for loss of clinical response and possible acquired resistance. In addition, co-administration of ritonavir in HIV patients may pose a risk of developing HIV-1 resistance to HIV protease inhibitors [62].

**Figure 1.**
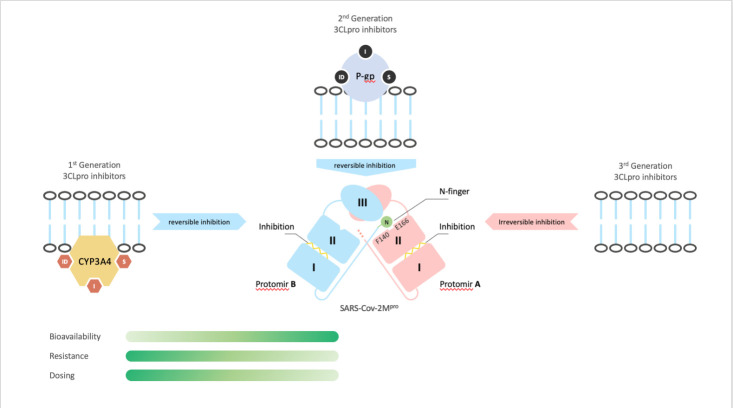
**Key differences in properties of 3CL protease inhibitors of SARS-CoV2**. The first generation is typically used in combination with a CYP3A4 inhibitor to achieve a sufficiently high plasma concentration of the active drug, which can lead to increased drug-drug interactions. Bioavailability is limited, requiring higher-than-desired doses, and resistance can develop rapidly in HIV patients. The second generation is assumed to be affected by P-gp interaction, which can also lead to resistance against new viral variants. The third generation holds potential for a single agent that acts irreversibly (in contrast to generations 1 and 2), has a single dose (high bioavailability), and less clinical resistance.

The second generation of novel 3CLpro inhibitors can probably achieve single agent clinical usage without a ritonavir boost, but the local concentration of the compounds may be reduced, which could impair inhibition by the multidrug P-gp transporter (Fig.1). Reduction of inhibitor concentration leads to viral mutant selection and possible acquired resistances, just as it was reported in anti-HIV-medication[63]. There are reported efforts to develop approaches to minimize the effect of P-gp in drug transport and to increase the bioavailability of the orally administered drug by use of P-gp inhibitors, specifically targeting P-gp efflux[64]. However, a single agent medication is always preferred in the real-word and clinical setting.

The third generation 3CLpro inhibitor could achieve single agent status in clinical usage without a ritonavir boost and inhibit the target in an irreversible manner. The inhibitory activity of a novel covalent irreversible core has not been impaired by the p-gp transporter. In addition, the third generation 3CLpro inhibitor could counter potential acquired viral resistance found in the clinic (Fig.1). The choice of administration route for a 3CLpro inhibitor needs to take into account multiple factors, including the timing of treatment, the stage of the COVID-19 illness, and the bioavailability of the antiviral. Currently, there is a need for a more convenient method of administering COVID-19 medication to early stage, non-hospitalized patients, as well as for pre-exposure and post-exposure prophylaxis options [65]. An ideal solution would be an oral delivery option for early-stage disease management or for pre-exposure/post-exposure use, at a high oral bioavailability (first-generation 3CLpro inhibitors PF-00835231 and GC-376 showed a bioavailability of 1.4% and 3%, respectively in rats). Third generation compounds hold a promise to meet these criteria [66]. As of February 2023, one compound of the third generation entered clinical trial stage. This is a covalent irreversible 3CLpro inhibitor developed with the assistance of artificial intelligence.

## Discussion

Although the rapid development of COVID-19 medications represents a significant success for science and public health, there are also some crucial limitations that should be addressed as the urgency of the pandemic subsides. First, the middle and long-term safety of these drugs could not be fully studied as they were repurposed or developed and tested within a shorter time frame than usual. Although clinical trials show that most of the drugs have a good safety profile, further monitoring and follow-up must be conducted to assess any potential long-term negative effects. Secondly, availability is also limited, as rapid development and approval do not automatically translate into broad availability. The production and distribution of these drugs remain a challenge and can lead to shortages and delays.

Thirdly, the effectiveness of these drugs against new virus variants is limited under the pressure of rapid development. As the virus constantly mutates, there is a possibility that the current medications may be less effective against newly emerging virus variants. These three aspects specifically affect the elderly population, as they are the most vulnerable ones to suffer from side effects and may not have equitable access to these medications.

Additionally, the economic burden of developing and producing these drugs is significant, which can result in high costs. This can pose challenges in financing and distributing vaccines, especially in resource-poor countries.

## Conclusion

COVID-19 infections affect vulnerable populations, with older adults experiencing a more severe disease course and worse prognosis due to biological aging. However, these older adults are often excluded from clinical trials, making safe and efficacious medication development a priority to avoid interaction with chronic diseases and therapies. Newly developed compounds can address several shortcomings of currently approved repurposed medications, including drug-drug interactions, limited effectiveness, reproductive risks, low safety profiles, and broad strains coverage. Second and third-generation 3CLpro inhibitors show promise in the development of effective treatments for SARS-CoV-2 infections. While antiviral protease kinase inhibitors were valuable during the acute pandemic setting, the priority in COVID-19 medication development should now be safe and selective therapies that do not harm the elderly. Further research is needed to fully assess the clinical efficacy and safety of these inhibitors, especially in mild/moderate cases in the outpatient setting. It is crucial to consider COVID-19 an infection that will continue to cause waves of infection, especially among vulnerable populations. With demographics sharply shifting towards negative growth, effective, minimally toxic, and elderly-suited medications are needed to mitigate the overall disease burden and sequelae of COVID-19, alongside prevention and advanced care planning. Tackling COVID-19 can also have an impact on healthy longevity by increasing disability-free life expectancy and improving the quality of life without significant limitations in daily activities. This study demonstrates the ongoing progress of medicine, as more than 50% of older adults require treatment for accompanying illnesses or risk factors in this age group.
